# Upregulated Renal Mineralocorticoid Receptor and 11β-Hydroxysteroid Dehydrogenase Type 2 Are Associated with K^+^ Retention During MR Antagonism in Female Spontaneously Hypertensive Rats

**DOI:** 10.3390/ijms27156953

**Published:** 2026-08-02

**Authors:** Irina Baranovskaya, Iryna Bilous, Sati Alexander, Bianca Tubalinal, Alena Cherezova, Vadym Buncha, Mikhail Fomin, Celso Gomez-Sanchez, Mykola Mamenko

**Affiliations:** 1 Immunology Center of Georgia, Augusta University, Augusta, GA 30912, USA; 2 Department of Physiology, Augusta University, Augusta, GA 30912, USA; 3 College of Allied Health Sciences, Augusta University, Augusta, GA 30912, USA; 4 Department of Physiology & Neuroscience, Keck School of Medicine, University of Southern California, Los Angeles, CA 90089, USA; 5 Department of Pharmacology and Toxicology, University of Mississippi Medical Center, Jackson, MS 39216, USA

**Keywords:** spontaneously hypertensive rat, 11β-hydroxysteroid dehydrogenase type 2, mineralocorticoid receptor, mineralocorticoid antagonism, hypertension, potassium, renin–angiotensin–aldosterone system

## Abstract

Mineralocorticoid receptor (MR) blockade exerts sex-specific effects in hypertension and cardiovascular disease; however, its role in blood pressure regulation in spontaneously hypertensive rats (SHRs) remains unclear. We assessed the effects of MR antagonism on blood pressure and electrolyte balance in male and female SHRs aged 10–15 weeks. Higher serum K^+^ levels in spironolactone-treated female SHRs were accompanied by transiently reduced urinary K^+^ excretion and a fourfold increase in aldosterone at the study’s end. These differences were not observed in male SHRs. Plasma renin activity and circulating corticosterone were 19% and 41% lower in females than in males but were unaffected by spironolactone in either sex. Plasma renin activity in SHRs was comparable to that of normotensive Wistar–Kyoto rats. Females exhibited approximately twofold higher renal MR and 1.7-fold greater renal 11β-hydroxysteroid dehydrogenase type 2 (11β-HSD2) protein levels. MR antagonism did not alter ENaC activity, α-ENaC protein abundance, blood pressure, or amiloride-sensitive urinary electrolyte excretion in either sex. Collectively, the upregulation of renal MR and 11β-HSD2 in females was associated with elevated circulating potassium in response to MR blockade, an effect that was likely counterbalanced by a compensatory rise in aldosterone. Low renin–angiotensin–aldosterone system activity in SHRs may limit the antihypertensive efficacy of MR antagonists.

## 1. Introduction

Mineralocorticoid receptor antagonists (MRAs) offer substantial clinical benefits in patients with primary aldosteronism, resistant and low renin hypertension, heart failure with reduced ejection fraction, type 2 diabetes and chronic kidney disease [1,2,3]. Yet, wider use of MRAs is limited by their adverse effects, such as the development of hyperkalemia. While all MRAs act as potassium-sparing diuretics, hyperkalemia becomes a serious concern, especially in patients with compromised renal function and concurrent use of renin–angiotensin–aldosterone system (RAAS) inhibitors [4,5]. The mechanisms accounting for the development of hyperkalemia in specific patient cohorts treated with MRAs are not entirely clear. Understanding these mechanisms is crucial for expanding the utilization of MRAs and developing targeted interventions with an improved safety profile.

A growing body of evidence reveals sex-specific differences in MR signaling, suggesting that biological sex may contribute to variability in responses to MRAs and affect the efficacy and safety of MR blockade. Clinical studies have shown that MRAs produce greater blood pressure (BP) reductions in women with salt-sensitive or resistant hypertension, including Black women, compared with men of the same race or hypertension subtype [6,7]. We and others have shown that MRAs effectively lower BP in female rats with AngII-dependent hypertension, Balb/C mice with salt-sensitive hypertension and agouti yellow obese female mice with leptin-induced hypertension, while the effect of MRAs in the respective male rodents was minimal [6,8,9,10]. One potential mechanism underlying these sex differences is the higher expression of MR and 11β-hydroxysteroid dehydrogenase type 2 (11β-HSD2) in target tissues from hypertensive female rodents, which may enhance aldosterone-dependent MR activation compared to males [8]. Further studies using animal models are needed to determine whether the differential responses to MRAs observed in males and females arise from distinct pathophysiological mechanisms or reflect a broader characteristic of hypertension and biological sex.

The spontaneously hypertensive rat (SHR) is a recognized rodent model, pivotal for studies on essential hypertension and its chronic cardiovascular effects [11]. The SHR model has been instrumental in elucidating genetic factors leading to essential hypertension, associated pathophysiological mechanisms and the efficacy of antihypertensive treatments. Numerous attempts have been made to characterize the activity of the RAAS in SHRs to collect somewhat controversial evidence. While the SHR is generally considered to have low-renin hypertension, the contribution of MR signaling to the increased BP and electrolyte homeostasis in SHRs remains to be fully understood and universally accepted.

This study aims to address these knowledge gaps and uses male and female SHRs to determine how MR blockade affects electrolyte homeostasis, BP, and key RAAS components. We assessed serum electrolytes, aldosterone, corticosterone, plasma renin activity (PRA), renal mRNA and protein expression of MR and 11β-HSD2, BP, renal epithelial sodium channel (ENaC) activity and αENaC subunit abundance, and urinary electrolyte excretion in 10–15-week-old SHRs treated with vehicle or spironolactone for 3 weeks. Age-matched Wistar–Kyoto (WKY) rats were used as normotensive controls where appropriate. Our findings indicate that greater renal abundance of MR and 11β-HSD2 in female SHRs promotes elevation of serum potassium in response to spironolactone, an effect that is ultimately offset by a compensatory rise in aldosterone. Low activity of the RAAS in SHRs of both sexes attenuates the antihypertensive effects of MRAs.

## 2. Results

### 2.1. Spironolactone Alters Potassium Homeostasis in Female SHRs but Not in Males

**Figure 1:**
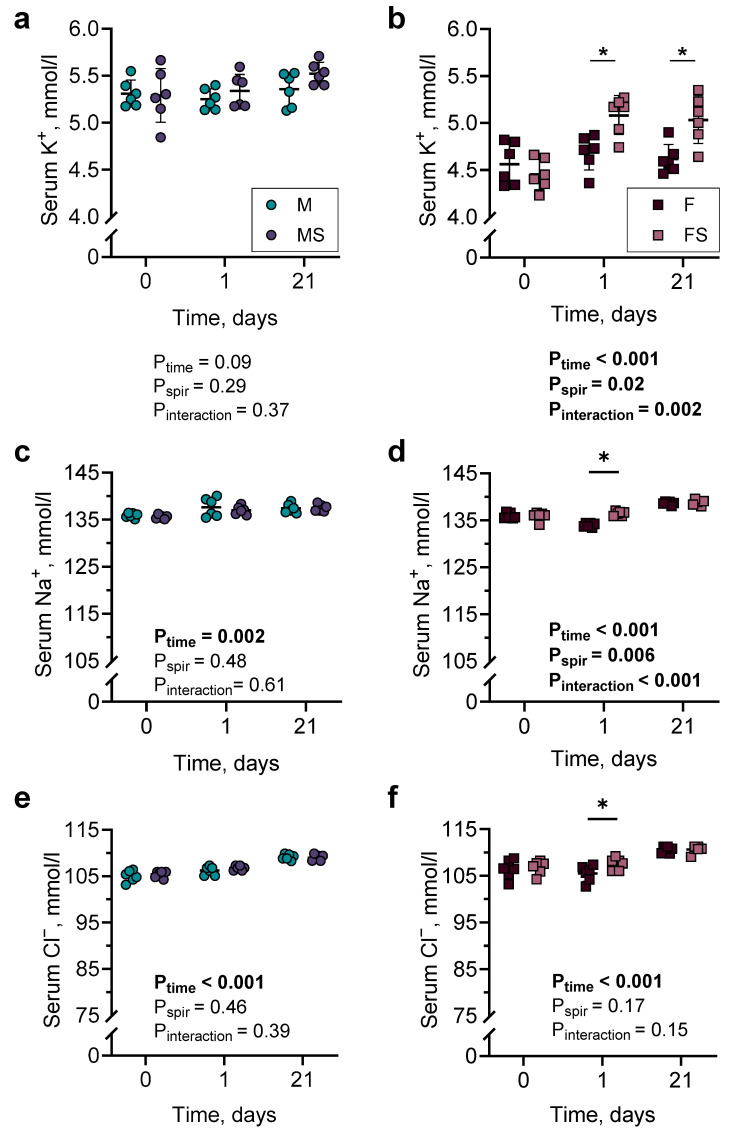
Spironolactone increases serum potassium in female but not male SHRs. Summary graphs showing serum K^+^, Na^+^, and Cl^−^ levels in male (**a**,**c**,**e**) and female (**b**,**d**,**f**) SHRs receiving vehicle (M, F) or spironolactone (MS, FS). Data were analyzed using repeated measures two-way ANOVA with Geisser–Greenhouse correction and Holm–Sidak post hoc test. *—denotes *p* < 0.05 for pairwise comparisons. Each group included *n* ≥ 6 rats.

First, we assessed the potassium-sparing effect of MR antagonism in male and female SHRs. Spironolactone (30 mg·kg^−1^·day^−1^) significantly increased circulating K^+^ levels in females from 4.45 ± 0.17 to 5.1 ± 0.20 mmol/L 24 h after the start of the treatment (Figure 1b). The endpoint serum K^+^ concentration remained significantly elevated (5.04 ± 0.26 mmol/L) in female SHRs after 3 weeks of spironolactone administration. In contrast, MR blockade did not alter serum K^+^ in male SHRs (Figure 1a), with K^+^ concentrations remaining in the 5.3–5.5 mmol/L range. SHRs of both sexes exhibited a modest (<5 mmol/L) rise in serum Na^+^ and Cl^−^ over the 3-week period (Figure 1c–f). Although these changes reached statistical significance, their physiological relevance appears minimal.

**Figure 2:**
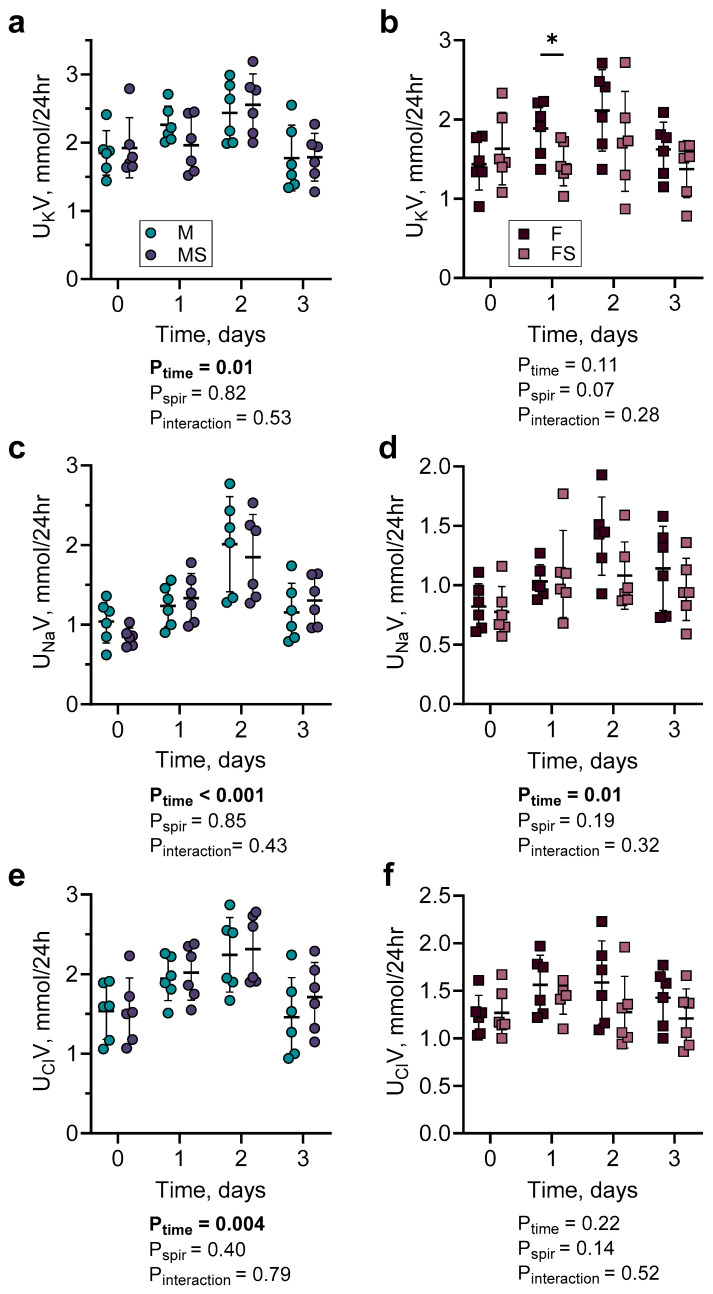
Sex-specific effects of spironolactone on urinary electrolyte excretion. Summary graphs showing urinary K^+^ (U_K_V), Na^+^ (U_Na_V) and Cl^−^ (U_Cl_V) excretion in male (**a**,**c**,**e**) and female (**b**,**d**,**f**) SHRs receiving vehicle (M, F) or spironolactone (MS, FS). Data were analyzed using repeated measures two-way ANOVA with Geisser–Greenhouse correction and Holm–Sidak post hoc test. *—denotes *p* < 0.05 for pairwise comparisons. Each group included n = 6 rats.

To determine whether the increase in serum K^+^ observed in female SHRs following spironolactone treatment was associated with reduced urinary potassium excretion (U_K_V), we analyzed 24-h urine samples collected from all experimental groups at baseline and during the first three days of treatment. We found that U_K_V was significantly lower in female SHRs on spironolactone than in vehicle-treated females on day 1 (1.43 ± 0.27 vs. 1.88 ± 0.35 mmol/24 h, respectively; *p* = 0.03; Figure 2b). A similar, although not statistically significant, trend was observed on days 2 and 3. In contrast, U_K_V did not differ significantly between vehicle- and spironolactone-treated male SHRs (Figure 2a). Spironolactone did not significantly alter urinary Na^+^ or Cl^−^ excretion in either male or female SHRs during the first three days of treatment (Figure 2c–f).

### 2.2. Circulating Aldosterone Is Significantly Increased in Spironolactone-Treated Female but Not in Male SHRs

**Figure 3:**
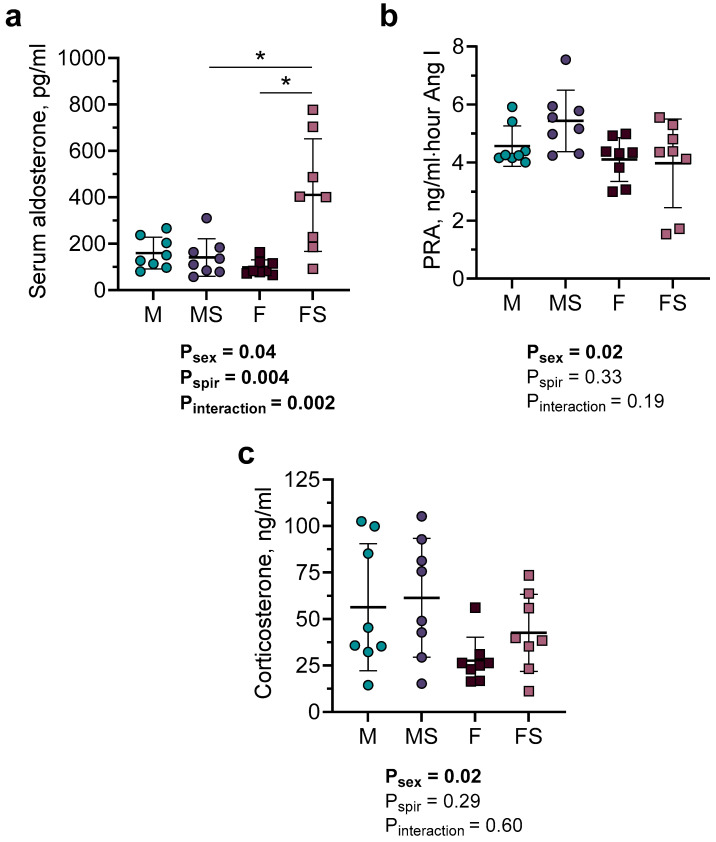
Spironolactone elevates circulating aldosterone in female SHRs, while PRA and serum corticosterone levels are lower in females compared to males. Summary graphs showing endpoint serum aldosterone concentrations (**a**), PRA (**b**) and serum corticosterone levels (**c**) in male and female SHRs. Data were analyzed using two-way ANOVA with the Holm–Sidak post hoc test. *—denotes *p* < 0.05 for pairwise comparisons. Each group included n ≥ 7 rats.

Given that elevated circulating K^+^ directly stimulates aldosterone secretion, we next measured serum aldosterone levels in SHRs. In vehicle-treated rats, endpoint aldosterone concentrations in both males and females were low and did not differ significantly (160 ± 69 and 98 ± 33 pg/mL, respectively; Figure 3a), comparable to levels in normotensive WKY rats (121 ± 84 and 108 ± 83 pg/mL in WKY males and females; Figure S1a). Spironolactone increased serum aldosterone approximately fourfold in female SHRs compared with sex-matched vehicle-treated controls (410 ± 242 vs. 98 ± 33 pg/mL), whereas no significant change was observed in males (140 ± 82 vs. 160 ± 69 pg/mL; Figure 3a). Endpoint PRA was modestly but significantly lower in female than in male SHRs (4.04 ± 0.09 vs. 5.01 ± 0.62 ng·mL^−1^·h^−1^ Ang I; Figure 3b). Overall, PRA in SHRs was similar to that in WKY rats (4.34 ± 0.74 vs. 4.78 ± 1.34 ng·mL^−1^·h^−1^ Ang I; Figure S1b). Spironolactone did not alter PRA in either sex. At the study endpoint, circulating corticosterone levels were significantly lower in female than in male SHRs (35 ± 18 vs. 59 ± 32 ng/mL) and were unaffected by spironolactone treatment (Figure 3c). These findings suggest that the marked increase in aldosterone observed in spironolactone-treated female SHRs is driven by elevated serum K^+^ to maintain potassium excretion, rather than by activation of the RAAS.

### 2.3. Renal Abundance of MR and 11β-HSD2 Is Higher in SHR Females

**Figure 4:**
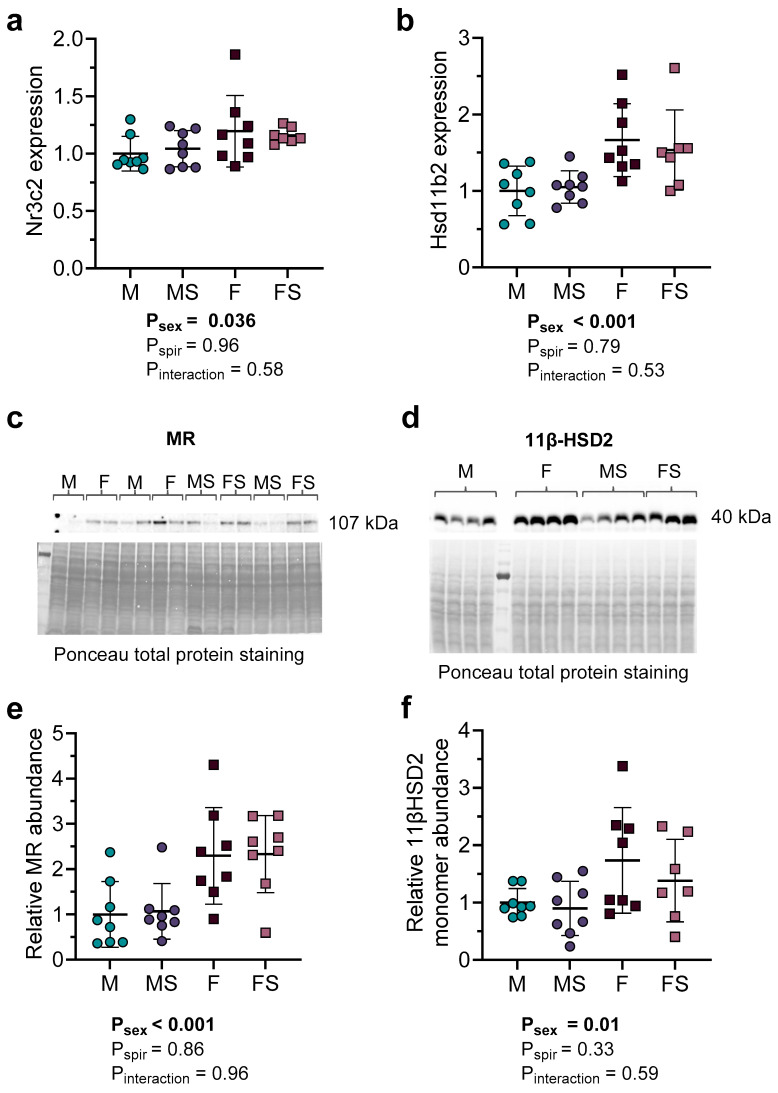
Elevated renal MR and 11β-HSD2 in female vs. male SHRs. (**a**,**b**) Relative renal expression of Nr3c2 mRNA, encoding MR (**a**), and Hsd11b2 mRNA, encoding 11β-HSD2 protein (**b**), in SHRs treated with vehicle (M, F) or spironolactone (MS, FS) normalized to Gapdh and expressed relative to the levels in vehicle-treated male SHRs. (**c**,**d**) Representative western blots showing the MR-reporting band at ~107 kDa (**c**), the 11β-HSD2-reporting band at ~40 kDa (**d**), and total protein loading (Ponceau red) in kidney homogenates from SHRs. (**e**,**f**) Quantification of relative renal MR (**e**) and 11β-HSD2 (**f**) abundance normalized to the total protein signal visualized using Ponceau S staining. Data were analyzed using two-way ANOVA with Holm–Sidak post hoc test. n ≥ 6 per group.

Greater responsiveness to MR blockade has been associated with higher renal MR expression in AngII-infused Sprague–Dawley female rats [8]. Accordingly, we assessed the relative expression of the nuclear receptor subfamily 3, group C, member 2 (Nr3c2) mRNA, encoding MR protein, as well as MR protein abundance in renal tissue homogenates from male and female SHRs. Renal Nr3c2 expression was marginally higher in female relative to male SHRs and was not significantly affected by spironolactone treatment (Figure 4a). In contrast, MR protein levels were approximately twofold higher in the kidneys of females relative to male SHRs (Figure 4c,e). MR binding by aldosterone or its antagonists depends on 11β-HSD2-mediated inactivation of corticosterone, which circulates at levels 100–1000-fold higher than aldosterone yet binds the MR with similar affinity. Both renal Hsd11b2 mRNA expression and 11β-HSD2 protein abundance were significantly higher in SHR females than in males (~1.4-fold for mRNA and ~1.7-fold for protein; Figure 4b,d,f). Higher levels of MR and 11β-HSD2 in SHR females increase MR availability to non-glucocorticoid ligands, such as aldosterone or spironolactone.

### 2.4. Aldosterone Antagonism Does Not Reduce BP in SHRs of Both Sexes

**Figure 5:**
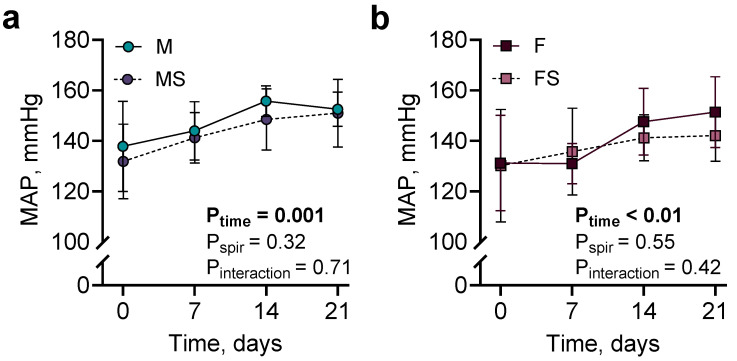
Spironolactone does not attenuate hypertension in SHRs. Mean arterial pressure (MAP) averages recorded by tail-cuff plethysmography at baseline, day 7, day 14 and day 21 in male (**a**) and female (**b**) SHRs treated with vehicle, M, F, or 30 mg·kg^−1^·day^−1^ spironolactone, MS, FS (n = 8 per group). Data were analyzed using repeated measures two-way ANOVA with the Geisser-Greenhouse correction. n = 8 per group.

Next, we assessed whether aldosterone antagonism had any systemic effects on arterial BP in SHRs. We found that administration of spironolactone at 30 mg·kg^−1^·day^−1^ did not result in any appreciable reduction of MAP in SHRs of both sexes (Figure 5a,b). After 3 weeks of treatment, MAP was 151 ± 13.4 and 142 ± 10.2 mmHg in spironolactone-treated male and female SHRs, respectively. This was not different from the MAP observed in vehicle-treated SHRs: 152 ± 6.8 and 151 ± 14.1 mmHg in males and females, respectively. Similarly, treatment with eplerenone (150 mg·kg^−1^·day^−1^), a more selective MRA with minimal affinity for androgen and progesterone receptors, did not significantly alter BP in SHRs of either sex (Figure S2a,b). After 3 weeks of treatment, MAP was 130 ± 5.4 and 128 ± 5.9 mmHg in vehicle- and eplerenone-treated males, and 129 ± 5.6 and 130 ± 5.3 mmHg in female SHRs receiving vehicle or eplerenone, respectively.

### 2.5. Spironolactone Did Not Affect the Endpoint ENaC Activity or Amiloride-Sensitive Urinary Electrolyte Excretion in SHRs of Both Sexes

**Figure 6:**
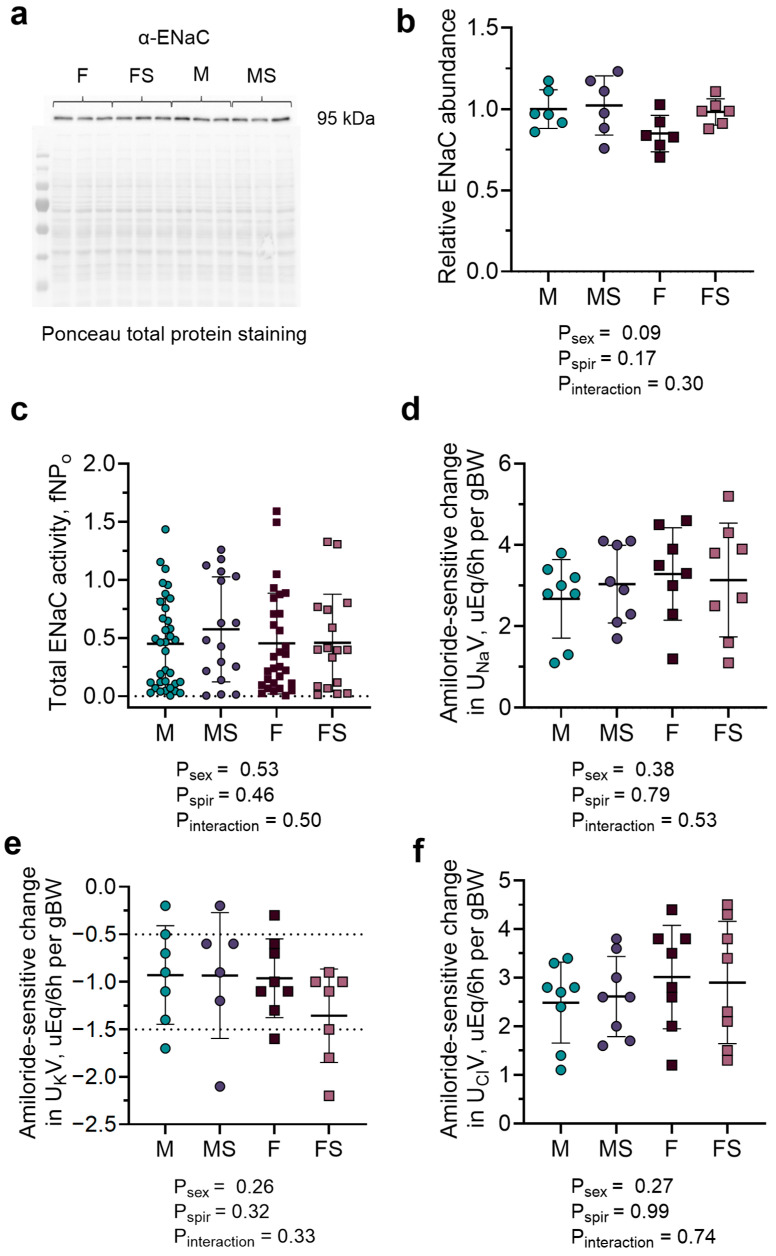
ENaC activity and amiloride-sensitive urinary electrolyte excretion are not affected by spironolactone administration in SHRs. (**a**) Representative western blot showing the α-ENaC-reporting band and total protein loading (Ponceau red) in kidney homogenates from male and female SHRs treated with vehicle (M, F) or spironolactone (MS, FS). (**b**) Quantification of the relative renal α-ENaC abundance in SHRs. (**c**) Total ENaC activity (fNP_o_) measured by patch-clamp in isolated cortical collecting duct segments. (**d**–**f**) Endpoint amiloride-sensitive urinary excretion of sodium, U_Na_V (**d**), potassium, U_K_V (**e**), and chloride, U_Cl_V (**f**), normalized to body weight. Data were analyzed using two-way ANOVA. n ≥ 6 rats per group.

Immunoblotting showed that 3 weeks of spironolactone treatment did not alter α-ENaC subunit abundance in renal tissue from male or female SHRs (Figure 6a,b). Patch-clamp recordings from split-open cortical collecting ducts revealed comparable levels of ENaC activity (fNP_o_ ≈ 0.4) in spironolactone- and vehicle-treated SHRs of both sexes (Figure 6c). Consistent with these findings, spironolactone had no effect on amiloride-sensitive urinary Na^+^, K^+^, or Cl^−^ excretion in either sex (Figure 6d–f).

## 3. Discussion

MRAs, particularly spironolactone, are established therapies for resistant hypertension, heart failure, and chronic kidney disease (CKD), conditions in which aldosterone–MR signaling contributes to disease progression. However, their broader clinical use is limited by safety concerns, most notably hyperkalemia in patients with impaired renal function or those receiving RAAS inhibitors.

Our study provides new insights into mechanisms that may influence MRA efficacy and safety by identifying sex-specific differences in responses to MR blockade in spontaneous hypertension. We found that in SHRs: (1) renal MR and 11β-HSD2 abundance is higher in females than in males; (2) spironolactone elevates serum K^+^ in females only; (3) females, but not males, exhibit a marked increase in circulating aldosterone following MR blockade; (4) females have lower PRA than males; (5) PRA activity in SHRs is comparable to that in normotensive WKY rats; (6) spironolactone did not affect the endpoint renal ENaC activity or amiloride-sensitive urinary electrolyte excretion; and (7) BP is unresponsive to MRA in both sexes. Overall, higher levels of renal MR and 11β-HSD2 in female SHRs increase MR availability for MRA. As a result, spironolactone acts as a potassium-sparing diuretic in females, promoting an increase in serum K^+^ that is counterbalanced by elevated aldosterone. However, MRA does not confer meaningful antihypertensive effects in young (10–15 weeks old) SHRs of either sex.

Our data suggest that higher renal 11β-HSD2 levels (Figure 4) determine the responsiveness of the kidney to MRAs in female SHRs compared to males. 11β-HSD2 inactivates corticosterone (or cortisol in humans), preventing it from binding to MR, which has equal affinity for corticosterone and aldosterone. When Hsd11b2 expression is low, MR is predominantly occupied by corticosterone due to its 100–1000-fold higher abundance [12]. Higher 11β-HSD2 abundance or activity reduces glucocorticoid occupancy of MR, enabling MRAs to compete mainly with aldosterone rather than glucocorticoids for receptor binding. As a result, lower MRA doses are sufficient to inhibit MR activation in tissues with high 11β-HSD2 activity [13]. The role of 11β-HSD2 is underscored by clinical observations in patients with apparent mineralocorticoid excess (AME) syndrome, associated with impaired HSD11B2 gene activity and manifesting in hypertension, where higher doses of MRAs are often required to normalize blood pressure [14]. Interestingly, women with salt-sensitive hypertension and hypertensive African American women reportedly benefit from MR antagonists more than men [6,7]. Our findings offer an unrecognized mechanistic explanation, suggesting that higher 11β-HSD2 abundance in females enhances their responsiveness to MRA therapy.

Another key observation from this study is that renal MR abundance is significantly higher in female SHRs than in males (Figure 4). We and others have demonstrated that aldosterone status cannot be interpreted independently of receptor availability and responsiveness [8,15,16]. Increased sensitivity to aldosterone may therefore exert a disproportionate effect on electrolyte balance. A critical gap remains in understanding how MR signaling in the distal nephron translates into variable physiological responses to aldosterone or MRAs. Our findings help address this gap by showing that enhanced renal MR abundance in female SHRs is associated with greater K^+^ retention in response to MRA.

Consistently, we found that urinary K^+^ excretion was lower in spironolactone-treated female SHRs than in vehicle-treated controls, resulting in elevated serum potassium levels in female SHRs on MRA, with no effect observed in males (Figure 1 and Figure 2). Mechanistically, MRAs inhibit potassium secretion in the distal nephron, promoting K^+^ retention and elevating serum K^+^ levels. This increase in serum potassium subsequently stimulates aldosterone secretion, thereby explaining the rise in aldosterone observed in spironolactone-treated female SHRs (Figure 3a). Renal outer medullary potassium (ROMK), transient receptor potential vanilloid 4 (TRPV4), and large-conductance calcium-activated potassium (BK) channels mediate potassium secretion in the distal nephron and are, at least in part, regulated by MR signaling [17,18,19]. However, an initial decrease in potassium channel abundance or activity induced by MRA can be offset by a compensatory elevation of circulating aldosterone. This appears to be the case in female SHRs at the study endpoint (day 21). Stabilized serum K^+^ levels in spironolactone-treated females (Figure 1) and the absence of differences in urinary K^+^ excretion after day 1 (Figure 2), as well as amiloride-sensitive potassium excretion between vehicle- and spironolactone-treated female SHRs (Figure 6), indicate a new homeostatic equilibrium. Identifying an earlier time point at which MRA reduces potassium channel abundance or activity, prior to the onset of aldosterone-mediated compensation, remains an important objective for future investigation.

Interestingly, baseline serum K^+^ levels were higher in male than in female SHRs (Figure 1). This finding is consistent with human and animal studies showing that males generally exhibit modestly higher serum K^+^ concentrations [20,21,22,23,24,25]. Potential contributors include sex differences in body composition, renal potassium handling, and mineralocorticoid signaling. Because approximately 98% of total body potassium is stored intracellularly, sex-dependent differences in potassium distribution may influence circulating K^+^ levels. Estrogen has been reported to enhance cellular potassium uptake and promote kaliuresis, whereas testosterone appears to exert anti-kaliuretic effects [26]. Together, these mechanisms may favor lower serum K^+^ concentrations in females and contribute to the higher baseline levels observed in male SHRs. For a comprehensive discussion of sex differences in potassium homeostasis, we refer readers to the review by Al-Qusairi [26].

A notable finding of the present study was that male SHRs exhibited higher circulating corticosterone levels and lower renal 11β-HSD2 abundance than females. This observation does not necessarily indicate enhanced activation of MR-dependent renal potassium transport by corticosterone in males. Clinically significant glucocorticoid-mediated MR activation associated with renal potassium wasting and hypokalemia is typically observed in states of marked (3–5-fold) glucocorticoid excess, such as ectopic ACTH syndrome, rather than in the setting of relatively modest elevations in circulating glucocorticoids [27]. We report a comparatively small (~1.7-fold) difference in corticosterone between male and female SHRs that was accompanied by approximately twofold lower renal MR abundance in males, a factor that would be expected to reduce the overall capacity for MR-dependent signaling. Furthermore, we found no evidence of enhanced activation of downstream sodium transport pathways in males, as ENaC expression and activity were similar between sexes. Collectively, these observations suggest that a modest increase in corticosterone and a reduction in renal 11β-HSD2 expression in male SHRs are insufficient to produce a meaningful increase in net renal MR signaling. Consequently, the absence of greater potassium wasting or hypokalemia in males should not be viewed as inconsistent with our findings.

We observed that PRA and aldosterone levels in vehicle-treated SHRs were comparable to those in normotensive WKY rats (Figure S1), indicative of a renin-independent, potassium-driven aldosterone response to MRA treatment. In line with blunted RAAS activity, spironolactone did not affect MAP, renal ENaC activity, αENaC subunit abundance, or amiloride-sensitive urinary Na^+^ excretion (Figure 5 and Figure 6). Likewise, administration of a more selective MR antagonist, eplerenone, failed to attenuate hypertension in SHRs of both sexes (Figure S2a,b), indicating that MR signaling does not significantly contribute to elevation of BP in SHRs. These observations are consistent with reports showing that SHRs exhibit low-renin hypertension, where elevated BP is primarily driven by increased sympathetic activity, endothelial dysfunction and vascular remodeling [28,29,30].

Nonetheless, several studies implicate renal aldosterone-MR signaling in the regulation of BP in SHRs. Transplantation of kidneys from SHRs into normotensive WKY rats elevates BP, whereas kidney transfer from WKY into SHR recipients attenuates hypertension [31,32]. Adrenalectomy prevents hypertension development in male SHRs, and this effect is reversed by aldosterone infusion [33]. Renal tissue from SHRs has been reported to exhibit increased ENaC expression and enhanced apical trafficking of aquaporin-2 compared to normotensive WKY rats, suggesting enhanced sodium reabsorption [34,35]. Pharmacologic MR blockade with high-dose spironolactone (200 mg·kg^−1^·day^−1^) has been shown to reduce systolic BP in SHRs [36]. Discrepancies regarding the contribution of the aldosterone-MR axis to hypertension in SHRs likely arise from variations in experimental design, including animal age, stress and associated adrenocorticotropic hormone levels (with an attempt to control for this with dexamethasone-morphine [37]), suprapharmacologic spironolactone dosing, and differences in colony origin. The dose of spironolactone used in the present study (30 mg·kg^−1^·day^−1^) falls within the commonly used range of 25–50 mg·kg^−1^·day^−1^ required to achieve effective MR blockade and measurable physiological outcomes in rats. Collectively, our study reinforces the prevailing consensus that hypertension in young SHRs (10–15 weeks), irrespective of sex, is largely independent of MR activation.

Interestingly, accumulating evidence implicates 11β-HSD2 in BP salt sensitivity. Kidney-specific deletion of Hsd11b2 in mice resulted in higher abundance of distal nephron sodium transporters, sodium-chloride cotransporter (NCC) and ENaC, compared to wild-type controls. Notably, spironolactone administration markedly reduces BP and decreases the abundance of the αENaC subunit and phosphorylated NCC in the kidney [38]. In contrast, we observed low ENaC activity in SHRs, comparable to levels reported in normotensive rodents maintained on regular sodium intake [39,40]. This may explain why MRAs had little effect on BP in SHRs. Although we did not directly assess the effect of amiloride on blood pressure in SHRs, it is reasonable to anticipate a similarly modest effect. However, any reduction in BP could be somewhat greater with amiloride, given its direct inhibition of ENaC rather than competition with corticosterone and aldosterone for MR binding. Another paper using mice with a brain-specific knockout of Hsd11b2 arrived at the conclusion that salt sensitivity in these mice was not attributable to impaired renal sodium excretion or volume expansion [41]. Moreover, MR blockade failed to prevent the salt-induced BP elevation in mice with Hsd11b2 haploinsufficiency, whereas glucocorticoid receptor antagonism effectively reduced BP [42]. In our study, lower renal 11β-HSD2 abundance in male SHRs was paralleled by higher basal circulating corticosterone levels. However, renal 11β-HSD2 is unlikely to substantially influence systemic glucocorticoid levels, as it primarily acts locally to protect MR from glucocorticoid activation. Notably, overall corticosterone levels in SHRs are similar to those in WKY rats [43], which exhibit only mild salt sensitivity [44,45]. Overall, while the existing data consistently demonstrates the inverse relationship between 11β-HSD2 activity and BP salt sensitivity, the antihypertensive efficacy of MRAs in the context of reduced or ablated 11βHSD2 activity is not universally established.

In SHRs, high salt intake exacerbates hypertension [46], but is not required for the development or maintenance of elevated BP. In our study, SHRs had persistently elevated BP despite being maintained on a diet with normal sodium content (0.2%). Notably, males, exhibiting lower renal 11β-HSD2 abundance, displayed moderately higher blood pressure than females. Further studies are warranted to clarify the role of 11β-HSD2 in mediating salt sensitivity of blood pressure in SHRs.

Our findings should be interpreted in light of several limitations of the current study. First, the experiments were conducted exclusively in young SHRs within a narrow age range, limiting the generalizability of the findings to animals at other stages of hypertension. Second, although female rats exhibited greater renal MR and 11β-HSD2 abundance that was associated with an enhanced potassium-retaining response to spironolactone, the evidence for a mechanistic role of these proteins is largely correlative. Direct causal approaches, such as pharmacological inhibition of 11β-HSD2, Nr3c2 or Hsd11b2 knockdown in the kidney, or the use of kidney-specific Hsd11b2-deficient models, were not employed. Third, the proposed model in which an initial MRA-induced reduction in renal potassium excretion is subsequently offset by compensatory increases in aldosterone requires further validation. Finally, our investigation focused on MR signaling and ENaC regulation and did not evaluate other renal potassium transport pathways that may contribute to sex-specific responses to MR blockade.

Future studies will investigate the causal roles of MR and 11β-HSD2 in mediating sex differences in potassium handling using targeted pharmacological and genetic approaches. Longitudinal experiments examining the temporal changes in ENaC activity, aldosterone production, and renal potassium excretion during MRA treatment would help clarify the compensatory mechanisms proposed in the present study. Additional work is also needed to evaluate the contribution of renal potassium transport pathways, including ROMK, BK, TRPV4 channels, and NCC-dependent mechanisms, to sex-specific responses to MR blockade. Finally, renal MR and 11β-HSD2 abundance, together with aldosterone and potassium levels during MR blockade, may represent promising mechanistic markers of MRA responsiveness for future translational studies.

In summary, our findings indicate that in young SHRs with low-renin hypertension, MR blockade does not effectively reduce BP but is associated with altered potassium balance in females. This effect is likely driven by greater renal abundance of 11β-HSD2 and MR, which enhances distal nephron sensitivity to MRA in female SHRs. The spironolactone-induced increase in serum potassium appears to be counterbalanced by a rise in aldosterone, establishing a new equilibrium. Clinically, our observations support consideration of sex as an important biological variable in MRA therapy, as females may be at greater risk of potassium retention despite comparable BP responses.

## 4. Materials and Methods

### 4.1. Experimental Rats

**Figure 7:**
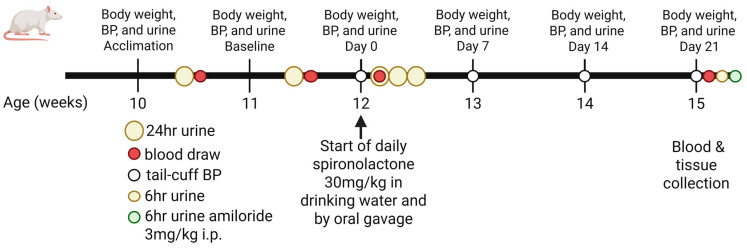
Experimental design and timeline.

All animal procedures were approved by the Institutional Animal Care and Use Committee of the Medical College of Georgia at Augusta University (AUP 2017-0844) and were conducted in accordance with the National Institutes of Health Guide for the Care and Use of Laboratory Animals. SHRs and WKY rats of both sexes purchased from Charles River had free access to a regular grain-based rodent chow (Teklad 2918) and tap water. Rats were maintained under conditions of constant temperature (+21 °C), humidity and a 12:12-h light–dark cycle throughout the study. The experimental timeline is presented in Figure 7.

### 4.2. Materials, Reagents and Supplies

All details on the materials, reagents and supplies used in this study, including vendors and catalog numbers, are provided in Table 1.

### 4.3. Assessment of Serum Electrolytes, Aldosterone, Corticosterone and PRA

On experimental days 1 and 21, blood samples (approximately 100–300 µL) were collected from anesthetized rats via the lateral tail vein using a 24 G needle and an intravenous catheter. Blood samples were collected into Microtainer serum separation tubes and allowed to clot at room temperature for 30 min, followed by centrifugation at 1500× *g* for 10 min to separate serum from blood cells. Serum electrolytes were analyzed using the Carelyte Plus ion-selective electrode system (Diamond Diagnostics, Holliston, MA, USA). At the experimental endpoint, trunk blood was obtained following decapitation for aldosterone, corticosterone and PRA assessment. To measure aldosterone and corticosterone, blood was collected into BD Vacutainer serum separation tubes, and serum was separated as described and stored at –80 °C. Endpoint aldosterone and corticosterone concentrations were measured in thawed serum samples using commercially available ELISA kits, following the manufacturer’s instructions. To assess endpoint PRA, blood was collected into tubes containing 5 mM EDTA at a 100:1 ratio. Samples were centrifuged at 1500× *g* for 15 min at 4 °C, and plasma aliquots were stored at −80 °C until analysis. PRA was measured in thawed plasma samples using a PRA ELISA kit, according to the manufacturer’s protocol. Absorbance was read at 450 nm using a µQuant Universal Microplate Spectrophotometer (Bio-Tek Instruments, Winooski, VT, USA). All serum and plasma samples were visually inspected for hemolysis. No samples exhibited significant hemolysis requiring exclusion.

### 4.4. Tissue Collection

At the end of the study, all rats were euthanized by decapitation under 5% isoflurane. The kidneys were immediately harvested for isolation of the collecting duct segments or quickly frozen in liquid nitrogen and stored at −80 °C for future use.

### 4.5. Quantitative Reverse Transcriptase Polymerase Chain Reaction (qRT-PCR)

Rat renal tissue was homogenized in 10 volumes of TRIzol reagent using a handheld Fisherbrand 150 homogenizer (OMNI International, Kennesaw, GA, USA). Total RNA extraction was performed according to the manufacturer’s protocol. The quality of the extracted RNA was verified by electrophoretic separation in a 1% agarose gel in TAE buffer (Tris-acetate-EDTA). Then, 1 μg of extracted RNA was digested with DNase followed by complementary DNA synthesis using the Reverse Transcriptase System according to the manufacturer’s protocols. Quantitative real-time PCR analysis was performed using iTaq Universal SYBR Green Supermix and the QuantStudio 3 RT-PCR System (Applied Biosystems by Thermo Fisher Scientific, Waltham, MA, USA). Primers were synthesized by Integrated DNA Technologies (Coralville, IA, USA) and provided in Table 1. The relative expression was calculated by the 2^−ΔΔCt^ method, and Gapdh mRNA was used for normalization. The average value of relative gene expression in the renal samples of untreated males (M) was used as ‘1.0’ for relative expression quantitation.

### 4.6. Immunoblotting

The renal tissue was homogenized in an ice-cold 5% sorbitol, 5 mM histidine/imidazole buffer (pH = 7.5) supplemented with a protease and phosphatase inhibitor cocktail using a handheld Fisherbrand 150 homogenizer (OMNI International, Kennesaw, GA, USA). The homogenates were centrifugated at 2000 *g* for 10 min at +4 °C, and protein concentration was measured using the Bradford assay with bovine serum albumin as a standard. Then, the samples were mixed with the Laemmli Sample Buffer containing 5% β-mercaptoethanol and incubated for 15 min at +60 °C. Samples were next separated by SDS PAGE using a 5/15% polyacrylamide gel at 110 V for 75 min and transferred to a nitrocellulose membrane using a Trans-Blot Turbo semidry transfer system (Bio-Rad Laboratories, Hercules, CA, USA) for 7 min at 25 V. To verify the amount of protein, the membrane was stained with Ponceau S using standard procedures. Membranes were blocked in 5% non-fat milk) in Tris-buffered saline with 0.1% Tween-20 (TBST) for 1 h at room temperature, followed by an overnight incubation at 4 °C with a polyclonal rabbit anti-rat antibody against the αENaC subunit (1:1000 dilution), a monoclonal mouse anti-rat antibody against MR (1:100 dilution) and a polyclonal sheep anti-rat antibody against 11β-HSD2 (1:5000 dilution). Then, the membranes were washed 3 times in TBST and incubated with secondary peroxidase-conjugated goat anti-rabbit (1:10,000), goat anti-mouse (1:5000) or donkey anti-sheep (1:2000) antibodies for 1 h at room temperature. After three washes with TBST, the membranes were incubated with a SuperSignal Pico Plus chemiluminescent kit and analyzed using the Azure 600 Imaging System (Azure Biosystems, Dublin, CA, USA). Densitometric analysis was performed in ImageJ 1.50 software (National Institutes of Health, Bethesda, MD, USA). The intensities of specific protein bands were normalized to the total protein signal according to the Ponceau S staining of the nitrocellulose membrane.

### 4.7. BP Assessment

To evaluate the antihypertensive effect of spironolactone on SHRs, BP was measured by tail-cuff plethysmography with the CODA acquisition system (Kent Scientific Corporation, Torrington, CT, USA) in lightly anesthetized (0.5–1% isoflurane) unrestrained rats, as described earlier [8]. In brief, baseline arterial BP was recorded in 10-week-old SHRs of both sexes after acclimation to the BP monitoring procedure. After that, SHRs were randomized into 4 groups: vehicle-treated males (M), vehicle-treated females (F), spironolactone-treated males (MS) and spironolactone-treated females (FS). Spironolactone was administered via oral gavage at a dose of 30 mg·kg^−1^·day^−1^ resuspended in tap water. Spironolactone-treated rats also received the drug in their drinking water while vehicle rats received tap water. Arterial BP was measured for two consecutive days at the beginning of each experimental week. To minimize circadian variations, BP was recorded from 10 a.m. to 12 p.m. The timeline and design for the experiments with eplerenone were identical. SHRs were randomized into the groups receiving vehicle (M, F) and a 150 mg·kg^−1^·day^−1^ dose of eplerenone (ME, FE). For all rats, BP data are presented as the MAP.

### 4.8. Single-Channel Recordings of ENaC Activity in Isolated Collecting Duct Segments

Collecting duct segments for patch-clamp recordings were isolated as described previously [8]. In brief, SHRs were euthanized with 5% isoflurane followed by exsanguination through renal arteries after the kidneys were collected. Excised kidneys were cut into thin (<1 mm) slices that were placed in an ice-cold bath solution containing (in mM) 150 NaCl, 5 KCl, 1 CaCl_2_, 2 MgCl_2_, 5 glucose and 10 HEPES (pH 7.35). Collecting duct segments were micro-dissected from the slices and split open to gain access to the apical membrane. ENaC channel activity was monitored in cell-attached patches under voltage clamp conditions at −V_p_ = −60 mV. Pipette solution contained (in mM): 140 LiCl, 2 MgCl_2_ and 10 HEPES (pH 7.35). Recordings were acquired with an Axopatch 200B amplifier interfaced to a computer running pClamp 10.6 software through a Digidata 1440 digitizer (Molecular Devices, San Jose, CA, USA). Collecting duct segments isolated from at least 5 rats were analyzed per experimental group.

### 4.9. Urinary Na^+^, K^+^, Cl^−^ Excretion

Twenty-four-hour urine samples were collected in metabolic cages on experimental days 0 (baseline), 1, 2, and 3 to compare Na^+^, K^+^ and Cl^−^ excretion in all SHR groups. To assess amiloride-sensitive renal Na^+^, K^+^, and Cl^−^ excretion at the study endpoint, we calculated the difference between urinary Na^+^, K^+^, and Cl^−^ excretion at baseline and after an intraperitoneal injection of 3 mg/kg amiloride hydrochloride dihydrate. Urine was collected in metabolic cages for 6 h on two consecutive days: (1) at baseline and (2) following amiloride injection. Rats were acclimated to the cages several times before the initiation of data collection. Urinary Na^+^, K^+^, and Cl^−^ concentrations were measured by the CareLyte Plus ion-selective electrode system (Diamond Diagnostics, Holliston, MA, USA) according to the manufacturer’s instructions. To calculate urinary Na^+^, K^+^, and Cl^−^ excretion, these values were multiplied by the urine volumes collected in metabolic cages over the course of 6 h for each animal and normalized to the animal’s body weight.

### 4.10. Statistical Analysis

Statistical analysis was performed in GraphPad Prism 10.6.1. The homogeneity of variances and the normality of distributions were determined using the Bartlett and Shapiro–Wilk tests, respectively. For repeated measurements such as serum electrolytes and BP, mixed-effects two-way ANOVA with the Geisser–Greenhouse correction was used. For other measurements two-way ANOVA was used. Holm–Sidak post hoc test was used when main effects or the interaction effect was found to be significant. A *p*-value of 0.05 was considered the threshold of significance. Data are presented as mean ± SD.

## Figures and Tables

**Table 1:** Major Resources.

**Primers for qRT-PCR**. Source: Integrated DNA Technologies (Coralville, IA, USA).				
**Target gene**	**Strand**	**Primer sequence (5′-3′)**	**Product length, bp**	
*Gapdh*	Forward	TGGGAAGCTGGTCATCAAC	78	
	Reverse	GCATCACCCCATTTGATGTT		
*Hsd11b2*	Forward	TGCTTGGCAGCCTATGGAAC	137	
	Reverse	ACATTAGTCACTGCCTCTGTCTTG		
*Nr3c2*	Forward	CTTTGGCAGTTTCCCAGTGC	152	
	Reverse	CACATTGCTCGCATGTACGG		
**Antibodies**				
**Description**		**Source**	**Catalog No.**	**Dilution**
Rabbit anti-rat ENaC alpha polyclonal antibody		StressMarq Biosciences (Victoria, British Columbia, Canada)	SPC-403	1:1000
Mouse anti-mineralocorticoid receptor monoclonal antibody		A gift from C. Gomez-Sanchez, University of Mississippi Medical Center (Jackson, MS, USA)	rMR1-18 1D5	1:100
Sheep anti-rat 11βHSD2 polyclonal antibody		A gift from C. Gomez-Sanchez, University of Mississippi Medical Center (Jackson, MS, USA)	2147	1:5000
Peroxidase-conjugated goat anti-rabbit IgG		Jackson ImmunoResearch Laboratories (West Grove, PA, USA)	111035144	1:10,000
Peroxidase-conjugated donkey anti-sheep IgG Antibody		R&D Systems Bio-Techne Corporation (Minneapolis, MN, USA)	HAF016	1:2000
Peroxidase-conjugated goat anti-Mouse IgG (H+L) antibody		Invitrogen (Carlsbad, CA, USA)	31430	1:5000
**Chemicals, supplies, consumables, ELISA kits**				
**Description**		**Source**	**Catalog No.**	
Spironolactone		TCI America (Portland, OR, USA)	RN 52-01-7	
Eplerenone		Sandoz (Basel, Switzerland)	NDC: 54868-6065	
Amiloride hydrochloride dihydrate		Alfa Aesar Thermo Scientific Chemicals (Ward Hill, MA, USA)	J62168	
24G needle and catheter		Covetrus (Portland, ME, USA)	070281	
Microtainer serum separation tubes		Beckton Dickinson (Franklin Lakes, NJ, USA)	365967	
Protease and phosphatase inhibitor cocktail		Thermo Scientific (Waltham, MA, USA)	A32959	
Non-Fat Dry Milk		Lab Scientific bioKEMIX (Danvers, MA, USA)	M0841	
SuperSignal West Pico Plus Chemiluminescent Substrate		Thermo Scientific (Waltham, MA, USA)	34577	
Nitrocellulose membrane		Bio-Rad Laboratories (Hercules, CA, USA)	1620115	
Trizol Reagent		Invitrogen (Carlsbad, CA, USA)	15596018	
RQ1 RNase-Free DNase		Promega Corporation (Madison, WI, USA)	M6101	
Reverse Transcriptase System		Promega Corporation (Madison, WI, USA)	A3500	
iTaq Universal SYBR Green Supermix		Bio-Rad Laboratories (Hercules, CA, USA)	1725121	
PRA ELISA kit		IBL America (Minneapolis, MN, USA)	IB59131	
Aldosterone ELISA kit		IBL America (Minneapolis, MN, USA)	IB79134	
Corticosterone ELISA kit		IBL America (Minneapolis, MN, USA)	IB79175	

## Data Availability

The original contributions presented in this study are included in the article and Supplementary Material. Further inquiries can be directed to the corresponding authors.

## Supplementary Materials

The following supporting information can be downloaded at https://www.mdpi.com/article/10.3390/ijms27156953/s1.

## Abbreviations

The following abbreviations are used in this manuscript:

ANOVA	Analysis of variance
BP	Blood pressure
ENaC	Epithelial sodium channel
Hsd11b2	A rat gene encoding 11β-hydroxysteroid dehydrogenase type 2
HSD11B2	A human gene encoding 11β-hydroxysteroid dehydrogenase type 2
11β-HSD2	11β-hydroxysteroid dehydrogenase type 2 (enzyme)
MAP	Mean arterial pressure
MR	Mineralocorticoid receptor
MRA	Mineralocorticoid receptor antagonist
NCC	Sodium-chloride cotransporter
PRA	Plasma renin activity
RAAS	Renin–angiotensin–aldosterone system
SHR	Spontaneously hypertensive rat
WKY	Wistar–Kyoto rat
