# Supplementary material for: Upregulated Renal Mineralocorticoid Receptor and 11β-Hydroxysteroid Dehydrogenase Type 2 Are Associated with K+ Retention During MR Antagonism in Female Spontaneously Hypertensive Rats
Source: Int J Mol Sci. 2026 Aug 2;27(15):6953. doi: 10.3390/ijms27156953 (PMC13467425; doi:10.3390/ijms27156953)
Supplement: Supplementary file 1 [file ijms-27-06953-s001.zip › ijms-4398888-supplementary.pdf]

International Journal of
Molecular Sciences

MDPI

Article

Upregulated Renal Mineralocorticoid Receptor and 11β-Hydroxysteroid Dehydrogenase Type 2 Are Associated with K^+ Retention During MR Antagonism in Female Spontaneously Hypertensive Rats

Irina Baranovskaya [1,*,†], Iryna Bilous [2,†], Sati Alexander [2], Bianca Tubalinal [3], Alena Cherezova [2], Vadym Buncha [4], Mikhail Fomin [1], Celso Gomez-Sanchez [5] and Mykola Mamenko [2,*]

1 Immunology Center of Georgia, Augusta University, Augusta, GA 30912, USA
2 Department of Physiology, Augusta University, Augusta, GA 30912, USA
3 College of Allied Health Sciences, Augusta University, Augusta, GA 30912, USA
4 Department of Physiology & Neuroscience, Keck School of Medicine, University of Southern California, Los Angeles, CA 90089, USA
5 Department of Pharmacology and Toxicology, University of Mississippi Medical Center, Jackson, MS 39216, USA
* Correspondence: ibaranovskaya@augusta.edu (I.B.); mmamenko@augusta.edu (M.M.)
† These authors contributed equally to this work.

Supplementary Materials

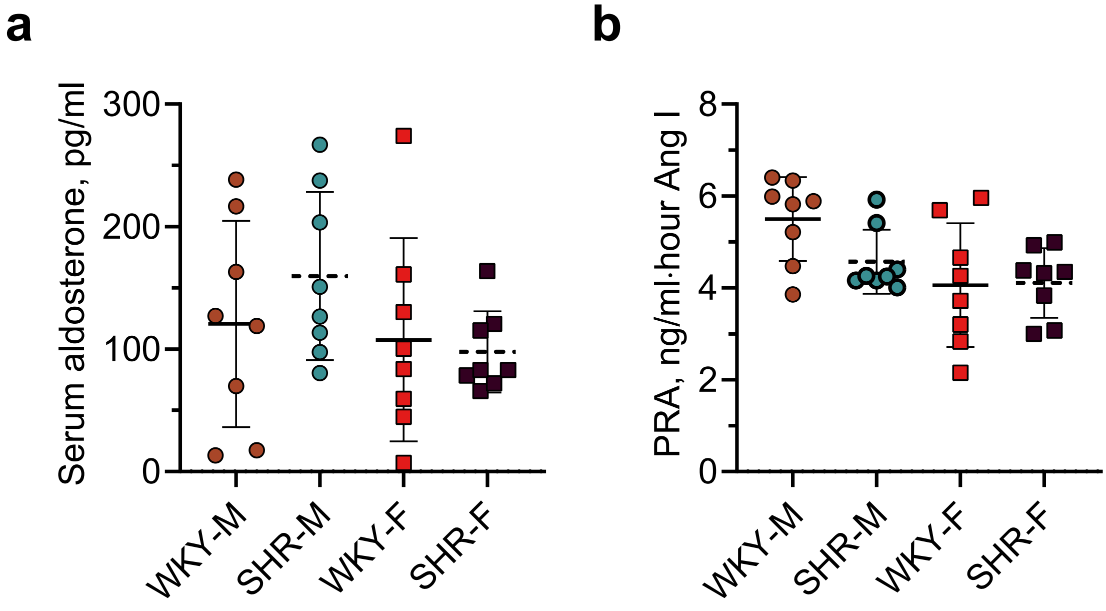

P_{sex} = 0.14
P_{strain} = 0.56
$P_{interaction}$ = 0.33

$\mathbf{P_{sex} = 0.009}$
P_{strain} = 0.21
$P_{interaction}$ = 0.16

Figure S1. The comparison of serum aldosterone levels and PRA in untreated SHR and WKY male and female rats. Differences were determined using two-way ANOVA. n = 8 per group.

 https://doi.org/10.3390/ijms27156953

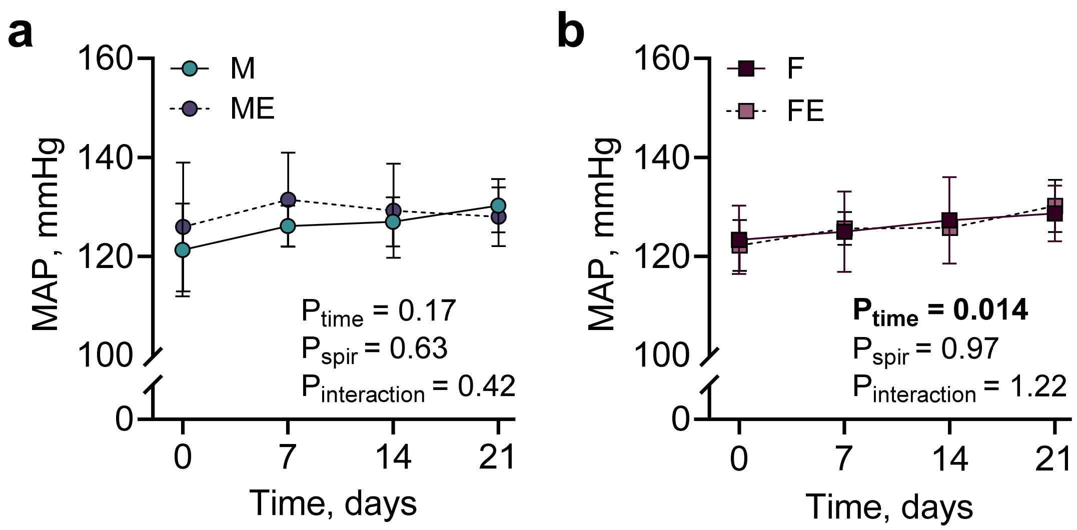

Figure S2. Eplerenone does not attenuate hypertension in SHR. Mean arterial pressure (MAP) averages recorded by tail-cuff plethysmography at baseline, day 7, day 14 and day 21 in male (a) and female (b) SHR treated with vehicle, M, F, or 150 mg·kg^{-1}·day^{-1} eplerenone, ME, FE (n=4 per group). Data were analyzed by repeated measures 2-way ANOVA with the Geisser-Greenhouse correction.

https://doi.org/10.3390/ijms27156953
